# Hypoxic Air Inhalation and Ischemia Interventions Both Elicit Preconditioning Which Attenuate Subsequent Cellular Stress *In vivo* Following Blood Flow Occlusion and Reperfusion

**DOI:** 10.3389/fphys.2017.00560

**Published:** 2017-08-02

**Authors:** James H. Barrington, Bryna C. R. Chrismas, Oliver R. Gibson, James Tuttle, J. Pegrum, S. Govilkar, Chindu Kabir, N. Giannakakis, F. Rayan, Z. Okasheh, A. Sanaullah, S Ng Man Sun, Oliver Pearce, Lee Taylor

**Affiliations:** ^1^Institute of Sport and Physical Activity Research, University of Bedfordshire Luton, United Kingdom; ^2^Sport Science Program, College of Arts and Sciences, Qatar University Doha, Qatar; ^3^Division of Sport, Health and Exercise Sciences, Department of Life Sciences, Centre for Human Performance, Exercise and Rehabilitation, Brunel University London Uxbridge, United Kingdom; ^4^Milton Keynes University Hospital Milton Keynes, United Kingdom; ^5^ASPETAR, Athlete Health and Performance Research Centre, Qatar Orthopedic and Sports Medicine Hospital Doha, Qatar; ^6^School of Sport, Exercise and Health Sciences. Loughborough University Loughborough, United Kingdom

**Keywords:** ischemia, oxidative stress, heat shock proteins, hypoxic preconditioning, ischemic preconditioning, knee surgery

## Abstract

Ischemic preconditioning (IPC) is valid technique which elicits reductions in femoral blood flow occlusion mediated reperfusion stress (oxidative stress, Hsp gene transcripts) within the systemic blood circulation and/or skeletal muscle. It is unknown whether systemic hypoxia, evoked by hypoxic preconditioning (HPC) has efficacy in priming the heat shock protein (Hsp) system thus reducing reperfusion stress following blood flow occlusion, in the same manner as IPC. The comparison between IPC and HPC being relevant as a preconditioning strategy prior to orthopedic surgery. In an independent group design, 18 healthy men were exposed to 40 min of (1) passive whole-body HPC (FiO_2_ = 0.143; no ischemia. *N* = 6), (2) IPC (FiO_2_ = 0.209; four bouts of 5 min ischemia and 5 min reperfusion. *n* = 6), or (3) rest (FiO_2_ = 0.209; no ischemia. *n* = 6). The interventions were administered 1 h prior to 30 min of tourniquet derived femoral blood flow occlusion and were followed by 2 h subsequent reperfusion. Systemic blood samples were taken pre- and post-intervention. Systemic blood and gastrocnemius skeletal muscle samples were obtained pre-, 15 min post- (15PoT) and 120 min (120PoT) post-tourniquet deflation. To determine the cellular stress response gastrocnemius and leukocyte Hsp72 mRNA and Hsp32 mRNA gene transcripts were determined by RT-qPCR. The plasma oxidative stress response (protein carbonyl, reduced glutathione/oxidized glutathione ratio) was measured utilizing commercially available kits. In comparison to control, at 15PoT a significant difference in gastrocnemius Hsp72 mRNA was seen in HPC (−1.93-fold; *p* = 0.007) and IPC (−1.97-fold; *p* = 0.006). No significant differences were observed in gastrocnemius Hsp32 and Hsp72 mRNA, leukocyte Hsp72 and Hsp32 mRNA, or oxidative stress markers (*p* > 0.05) between HPC and IPC. HPC provided near identical amelioration of blood flow occlusion mediated gastrocnemius stress response (Hsp72 mRNA), compared to an established IPC protocol. This was seen independent of changes in systemic oxidative stress, which likely explains the absence of change in Hsp32 mRNA transcripts within leukocytes and the gastrocnemius. Both the established IPC and novel HPC interventions facilitate a priming of the skeletal muscle, but not leukocyte, Hsp system prior to femoral blood flow occlusion. This response demonstrates a localized tissue specific adaptation which may ameliorate reperfusion stress.

## Introduction

Tourniquets are used in several surgical procedures (Fitzgibbons et al., 2012). Relative to total knee replacement (TKR) surgery their use facilitates a near bloodless field, improving visualization of crucial structures, and accelerating the surgical procedure (Smith and Hing, 2010; Estebe et al., 2011). However, the benefits of tourniquet application are not without negative post-surgical side-effects e.g., delayed wound healing, vascular injury, muscular damage, and greater post-operative pain (Estebe et al., 2011; Fitzgibbons et al., 2012). Tourniquet mediated side-effects are not restricted to solely tissue damage (via direct tourniquet tissue compression), but also through ischemic reperfusion (IR) mediated metabolic disruption (Fitzgibbons et al., 2012). Reperfusion of occluded tissue (i.e., the leg after TKR specific tourniquet use) may lead to ischemic injury with activated leukocytes increasing reactive oxygen species (ROS) and/or free-radical formation, consequently resulting in transient elevations in oxidative stress (OS, Grace, 1994). Although ROS are essential for normal cellular signaling (Ray et al., 2012) and demonstrate an association with hormesis (Radak et al., 2008), a rapid increase in ROS can disrupt redox balance, resulting in OS induced protein denaturation and lipid oxidation (Halliwell and Chirico, 1993; Hawkins and Davies, 2001).

Reduced glutathione (GSH) is the most abundant endogenous antioxidant and can acquiesce increases in OS via its oxidation (e.g., cysteine originated reducing equivalent donation of H^+^+ e^−^ to unstable molecules, such as, ROS; i.e., ROS scavenging) to oxidized glutathione (GSSG). The GSH:GSSG ratio can indicate cellular redox balance, with increases in the latter indicative of increased OS and a pro-oxidant state (Fisher-Wellman and Bloomer, 2009). During knee prosthesis implantation surgery, following ~85 min tourniquet application (thigh), both local (the occluded leg), and systemic (the arm) blood borne free radical generators hypoxanthine and xanthine oxidase increased, whilst GSH:GSSG only demonstrated significant pro-oxidant values locally, all of which were seen 5 min post-reperfusion (Karg et al., 1997). Elevated OS following IR of tissues is associated with inflammation and impaired wound healing (Soneja et al., 2005), and postulated to induce greater post-operative pain (Orban et al., 2006), whilst acquiescing OS reduces oxidant mediated apoptosis (Primeau et al., 2002) and post-operative pain (Waikakul et al., 1999). Interestingly, short non-lethal cycles of IR [ischemic preconditioning (IPC)] can prime the intended tissue and bestow protection for future IR stress (Murry et al., 1986; Saita et al., 2002). This method of IR has been shown to reduce knee surgery mediated increases in OS following tourniquet use when compared to controls (Koca et al., 2011). Furthermore, IPC *in vivo* has been shown as effective in reducing post-operative knee surgery pain and/or corresponding length of hospital stay (Memtsoudis et al., 2010).

Inhaled hypoxic air preconditioning (HPC) can infer cellular tolerance to subsequent hypoxia-mediated OS *in vivo* (Taylor et al., 2012), inducing similar protective effects to those conveyed by IPC (Bushell et al., 2002a,b; Berger et al., 2010; Samaja and Milano, 2015; Verges et al., 2015; Chacaroun et al., 2017), with the simplicity of the intervention (i.e., inhaling hypoxic air) advantageous compared to IPC. At present HPC, unlike IPC, has not been utilized prior to TKR to alleviate IR stress *in vivo*, despite similar protective effects. Characterization of occlusion mediated blood and muscle IR stress responses are heavily biased to OS markers. Given cells can initiate protective mechanisms during OS complimentary to GSH reduction, particularly increasing transcription and translation of a highly conserved cytoprotective family of proteins, known as heat shock proteins (HSPs) (Kalmar and Greensmith, 2009; Morton et al., 2009b), characterization of both OS and HSP responses to TKR like occlusion IR stress appears mechanistically warranted. Indeed, *in vivo* HPC mediated increases in basal HSP72 and hemeoxygenase-1 (HSP32) were both associated with resisting subsequent hypoxia/reoxygenation (HReox, Samaja and Milano, 2015) induced OS upon systemic HReox; attributed to restoring the function of OS mediated denatured proteins (principally HSP72, Taylor et al., 2010a, 2012) and degradation of ROS-producing heme molecules (principally HSP32, Gozzelino et al., 2010; Taylor et al., 2012). Readers are directed to multiple reviews for a detailed overview of the transcription factors and functional roles of HSP72 and HSP32 (Kregel, 2002; Morton et al., 2009b; Gozzelino et al., 2010; Henstridge et al., 2014).

Contextually, the cost of TKR surgery is ~$7,500 (Dakin et al., 2012), with the majority of this cost associated with long duration post-operative patient hospitalization (Smith et al., 2008). IPC has some efficacy for positively influencing variables associated with post-operative length of stay, however its administration typically occurs within the operating theater, and thus is not economically viable regarding time (theater availability is finite and often pressurized, operating list congestion, etc.). Therefore, a pre-operative (ideally on the ward) easily administrable preconditioning intervention (i.e., HPC) with positive physiological effects akin to IPC would be advantageous clinically (improved surgical outcome) and financially (reduced post-operative length of stay without increased time within theater). Logistically, HPC may be facilitated by supplementary low O_2_ gas inhalation in an equivalent manner to that of supplementary O_2_ or medical Nitrous oxide (Berkowitz et al., 1976; Greif et al., 2000), or via inhalation of nitrogen rich gas administered through small portable generators utilized in athletic training (Millet et al., 2010) or by creating rooms of low O_2_ concentration via nitrogen gas (Mekjavic et al., 2016; Simpson et al., 2016). Given acute exercise elicits significant OS across populations and modalities (Fatouros et al., 2004; Vincent et al., 2005; Hillman et al., 2011; Taylor et al., 2016), with this increase impacting muscle force production (Powers and Jackson, 2008), the benefits of HPC and IPC as a preconditioning strategies to ameliorate the cellular disruptions to contractile function in this paradigm also warrant further investigation. Physical activity (Debevec et al., 2017) and exercise training reduce OS (Miyazaki et al., 2001; Vinetti et al., 2015), with training induced elevations in intramuscular HSP content (Liu et al., 1999; Morton et al., 2009a) a potential pathway for these benefits (Oksala et al., 2014). As such HPC (and IPC) may provide an alternative strategy for individuals undertaking exercise for health, or athletic performance to reduce the potentially negative impact of acute OS (Braakhuis and Hopkins, 2015; Vinetti et al., 2015).

Therefore, the aim of this study was to examine the effects of interventional HPC and IPC prior to subsequent TKR like tourniquet induced IR stress, in comparison to a control condition using an ecologically valid model. It is hypothesized that a bout of either HPC or IPC would prime the HSP system, providing resistance to the physiological stresses induced via tourniquet ischemia. Furthermore, tourniquet mediated stress would be monitored via associated redox markers (GSH/GSSG, protein oxidation) post-occlusion.

## Methods

### Ethical approval

The protocol was ethically approved by the University of Bedfordshire's Sport and Exercise Science Departmental Human Ethics Committee and all participants signed informed consent in accordance with the ethical standards outlined in the 1964 Declaration of Helsinki.

### Participants and general experimental controls

Eighteen apparently healthy male participants (see Table 1 for participant characteristics) volunteered and were subsequently randomly allocated to either control (CON), or an HPC intervention (HPC_I_) or an IPC intervention (IPC_I_). A standardized meal [cornflakes (50 g), milk (250 mL), and 1 l of water], as employed by others (Foster et al., 2016), was utilized within the experimental design. Relative to HSP and OS outcome variables, their between- and within-subject variation is established at rest (Fisher-Wellman and Bloomer, 2009; Sandström et al., 2009; Taylor et al., 2010b) and in response to stressors (Hillman et al., 2011; Lee et al., 2014; Peart et al., 2015). Accordingly an array of robust and previously employed experimental controls were incorporated within the study design to control for the confounding influences on HSP and/or OS responses of smoking (Anbarasi et al., 2006), caffeine (Whitham et al., 2006), glutamine (Wischmeyer et al., 2001; Zuhl et al., 2015), alcohol (Wu and Cederbaum, 2003), dietary consumption (Kuennen et al., 2011; Marshall et al., 2017), fluid intake (attainment of euhydration; Logan-Sprenger et al., 2015), generic supplementation (Pingitore et al., 2015), prior exercise (Lee et al., 2014), previous environmental (hypoxia and heat) exposures (Gibson et al., 2015b; Lee et al., 2016), and diurnal variation in basal HSP (Taylor et al., 2010a,b, 2011, 2012; Hillman et al., 2011; Costa et al., 2012). Apparent compliance was confirmed in 100% of participants and was monitored via a questionnaire prior to each experimental visit (e.g., CON, HPC_I_, or IPC_I_).

**Table 1:** Participant characteristic data.

**Measure**	**CON (*****n*** = **6)**			**HPC**_**I**_**(*****n*** = **6)**			**IPC**_**I**_**(*****n*** = **6)**		
	**Mean**	***SD***	**Range**	**Mean**	***SD***	**Range**	**Mean**	***SD***	**Range**
Age (years)	22.2	2.9	18–26	20.8	2.4	19–25	18.5^*^	0.6	18–19
Height (m)	1.83	0.06	1.75–1.92	1.77	0.10	1.67–1.93	1.79	0.04	1.73–1.83
Mass (kg)	80.4	12.4	62.8–93.4	73.5	8.7	61.9–86.6	76.7	7.8	64.8–87.4
Lean mass (%)	85.8	3.5	79.3–88.5	84.9	5.3	78.9–91.4	86.5	3.0	81.5–90.1
Fat mass (%)	14.2	3.5	11.5–20.7	15.3	5.5	8.6–22.1	13.5	3.0	9.9–18.5
Thigh circumference (cm)	44.7	2.6	40–47	42.8	2.1	40–46	43.3	2.4	39–46
Systolic blood pressure (mmHg)	124.0	3.0	120–129	125.8	1.9	123–129	125.5	2.1	123–129
Diastolic blood pressure (mmHg)	76.2	7.0	65–83	75.0	6.0	65–81	79.7	8.0	70–92

* *Significant difference vs. CON (p < 0.05)*.

Tourniquet applications throughout all relevant experimental procedures were produced via a straight 10 cm wide tourniquet cuff (AET, Anetic Aid, Leeds, UK) positioned superiorly to cotton wool padding on the thigh of the right leg, with pressure maintained by means of an electronic tourniquet unit (AET, Anetic Aid, Leeds, UK). During TKR this tourniquet pressure facilitates a bloodless field while minimizing direct compression injury (Worland et al., 1997).

### Experimental design

Participants arrived to the laboratory at 08:30 in a fasted stated (from at least 00:00 to arrival) and consumed the standardized meal for breakfast (this meal was provided again at 13:30 for lunch) and had anthropometric data collected. Blood pressure was recorded at baseline using an aneroid sphygmomanometer. They then rested within the laboratory under standardized environmental conditions until 11:30. At 11:30 participants were positioned in an inclined supine position for the remainder of the experimental protocol. Subsequently, participants rested for 1 h (until 12:30) prior to their 40 min allocated preconditioning intervention (HPC_I_or IPC_I_), with CON receiving an extended rest period.

#### HPC_I_

*HPC*_I_ participants inhaled ~14.3% O_2_ (simulated altitude of 2,980 m above sea level) for 40 min (from 12:30 until 13:10) in normobaric pressure via an adjustable hypoxicator (Everest Summit II, The Altitude Centre, UK) which produced the necessary hypoxic load via O_2_ filtration. Heart rate (HR; b.min^−1^) and peripheral oxyhaemoglobin saturation (SpO_2_; %) were measured every 5 min via finger pulse oximetry (Onyx® II 9550, Nonin Medical, USA) throughout the HPC_*I*_ intervention.

#### IPC_I_

*IPC*_I_ received four cycles of 5 min ischemia and 5 min reperfusion (total of 40 min from 12:30 until 13:10) at 100 mmHg above the participant's systolic pressure on their right leg in line with previous research (Koca et al., 2011).

**Figure 1:**
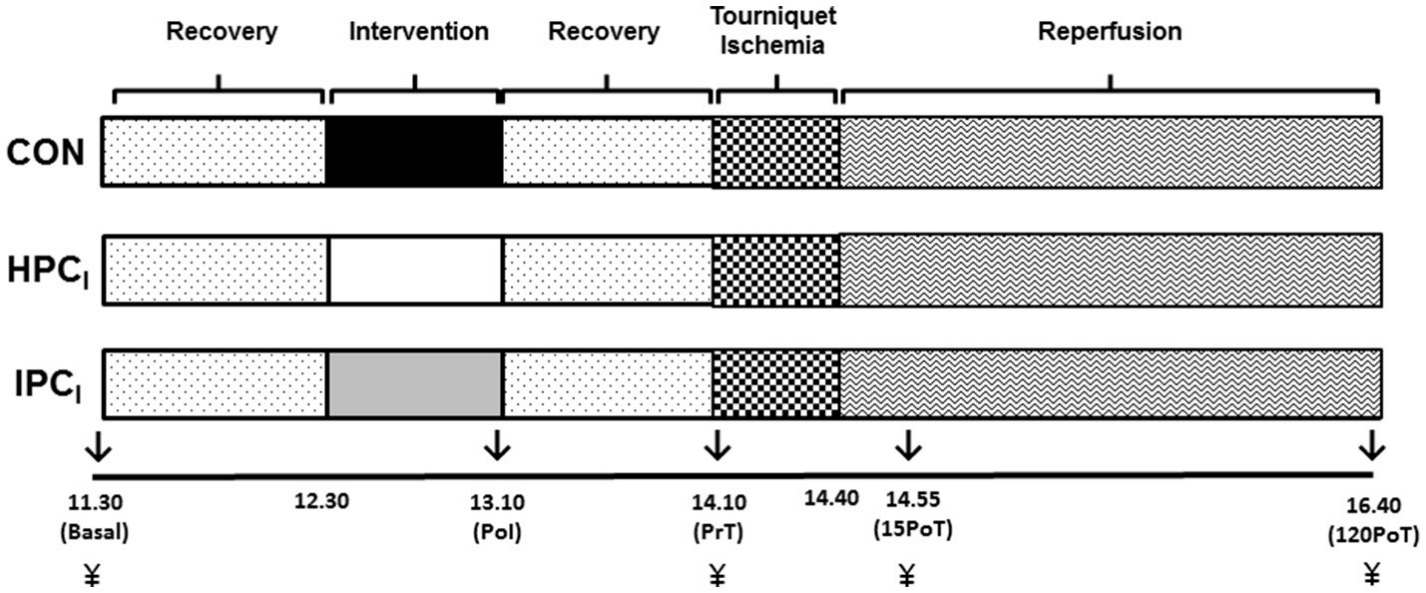
Experimental overview blood samples (↓) were obtained at basal, immediately post-intervention (PoI), immediately pre-tourniquet application (PrT), 15 min post-tourniquet removal (15PoT), and 120 min post-tourniquet removal (120PoT) with gastrocnemius tissue (¥) collected at PrT, 15PoT, and 120 PoT.

Upon cessation of the preconditioning intervention (HPC_I_ or IPC_I_) or CON (13:10), participants rested again for 55 min (14:05) prior to their right leg being elevated at 45° for 5 min (14:05–14:10), immediately followed by 30 min (14:10–14:40) tourniquet application, and subsequent 2 h (14:40–16:40) period of reperfusion (Figure 1).

Sample size calculations were determined via G.Power 3.1, (Universität Dusseldorf, Germany) (Faul et al., 2009) using data describing changes in Hsp72 mRNA from a publication external to our group (Mestre-Alfaro et al., 2012). For a two tailed test with an alpha of 0.05 and power of 0.80, it was calculated that six participants were required to find an Hsp72 mRNA increase of 3.8-fold significant. This sample size is ≥others in the field (Puntschart et al., 1996; Febbraio and Koukoulas, 2000; Fehrenbach and Northoff, 2001; Fehrenbach et al., 2003b; Liu et al., 2004; Mee et al., 2016).

### Blood sampling

#### Preparation for initial storage

Venous blood was drawn from an antecubital vein into three separate 4 mL Vacuette tubes (Vacuette®, Grenier Bio-One, UK) treated with either K_3_EDTA (Hsp), sodium citrate (glutathione) or lithium heparin [protein carbonyl (PC)] with all tubes filled to capacity. Blood samples were obtained (see Figure 1) at basal and immediately post-intervention (PoI), whilst also immediately pre-tourniquet application (PrT), 15 min post-tourniquet removal (15PoT), and 120 min post-tourniquet removal (120PoT) (Figure 1).

##### Leukocyte isolation for HSP analysis

Blood treated with K_3_EDTA underwent leukocyte isolation utilizing a previously validated technique (Tuttle et al., 2015; Gibson et al., 2015a,b; Mee et al., 2016). Briefly, 1 mL of K_3_EDTA blood was added to 1:10 red blood lysis solution (Miltenyi Biotec, UK) and allowed to incubate at room temperature for 15 min, prior to isolation via centrifugation at 400 × g for 5 min at 4°C. Supernatant was removed and the remaining pellet was washed twice in 2 mL of PBS solution (Fisher Scientific, UK) at 400 × g for 5 min at 4°C. The pellet was suspended in 1 mL of PBS and separated equally into two 1.5 mL RNase free microtubes (ThermoFisher Scientific, UK) then centrifuged at 17,000 × g for 5 min at 4°C. The remaining supernatant was aspirated prior to the pellet being completely re-suspended in 200 μL of TRIzol reagent (Sigma Aldrich, Dorset, UK) and stored at −80°C for subsequent RNA extraction.

##### Glutathione blood samples

Two milliliters of sodium citrate treated blood was immediately added to 8 mL of freshly prepared 5% metaphosphoric acid (Sigma Aldrich, Dorset, UK) and left to incubate on ice for 15 min prior to centrifugation at 12,000 × g for 15 min at 4°C. The clarified supernatant was collected and stored at −80°C until future analysis for total glutathione and GSSG utilizing commercially available kits [Glutathione (Total) Detection Kit, ADI-900-160, Enzo Life Sciences, Exeter, UK].

##### Protein carbonyl blood samples

A full 4 mL lithium heparinized blood tube was immediately centrifuged at 900 × g for 10 min at 4°C before the plasma was collected and stored at −80°C for future determination of PC concentration utilizing commercially available kits (Protein Carbonyl Colorimetric Assay Kit, 10005020, Caymen Chemical Company, Michigan, USA).

### Blood oxidative stress markers

#### Whole-blood glutathione

To determine the concentration of total glutathione, previously obtained supernatant (50 μL) was diluted to 1:40 with assay buffer solution and transferred to a 96-well plate in accordance with the manufacturer's instructions. A standard curve was created through serially diluting 50 μL GSSG standard and 50 μL of assay buffer solution (100–12.5 pmol). A 150 μL mixture of DTNB (5,5′-dithio-bis-2-nitrobenzoic acid) and 10 μL glutathione reductase was added to all wells to produce TNB (5-thio-2-nitrobenzoic acid) and immediately assessed via a microplate reader (Sunrise™, Tecan, Reading, UK) at an absorbance of 405 nm every minute for 10 min. For determination of GSSG, the method outlined above was replicated with the addition of samples first being treated with 1 μL of 2 M 4-Vinylpyridine (Sigma Aldrich, Dorset, UK) to block any free thiols from cycling the reaction. Four microliters of 2 M 4-Vinylpyridine was added to 200 μL of GSSG standard to produce a standard curve. Samples and standards were incubated for 1 h and analyses were identical to the protocol for total glutathione. GSH was calculated via subtraction of GSSG concentrations from total glutathione and a final GSH/GSSG ratio was computed. All standards and samples were run in triplicate and an average was taken. The intra- and inter-coefficient of variance for the assay kits were 3.4 and 3.6%, respectively, in line with previous research (Taylor et al., 2012).

#### Protein carbonyl

Plasma (200 μL) was added to 800 μL of 2,4-dinitrophenylhydrazine acting as the sample tube whilst 200 μL of plasma was added to 800 μL of 2.5 M hydrochloric acid to serve as the control tube. All tubes were required to incubate in the dark for 1 h at room temperature with a brief vortex every 15 min. One milliliter of 20% trichloroacetic acid was added to each tube, briefly vortexed and incubated on ice for 5 min prior to centrifugation at 10,000 × g for 10 min at 4°C. This was followed by a 10% trichloroacetic acid wash, incubation on ice for 5 min and centrifuged at 10,000 × g for 10 min at 4°C. Supernatant was discarded and the pellet suspended in a 1:1 ethanol/ethyl acetate wash before undergoing a thorough vortex and centrifugation at 10,000 × g for 10 min at 4°C. This was repeated twice more before the pellet was re-suspended in 500 μL of guanidine hydrochloride and centrifuged at 10,000 × g for 10 min at 4°C. An aliquot of 220 μL of both sample and control was added to a 96-well plate and the absorbance was measured at 360 nm using a microplate reader (Sunrise™, Tecan, Reading, UK). Calculation of PC concentration was determined following the manufacturer's instructions. All samples and standards were analyzed in duplicate. The intra and inter-assay coefficient of variance are 4.7 and 8.5%, respectively.

### Muscle biopsies

#### Muscle biopsy technique and preparation for initial storage

All biopsies were taken by medically qualified Orthopedic Surgeons, with full UK General Medical Council registration. Muscle biopsies were obtained using a previously validated and HSP specific *in vivo* technique (Morton et al., 2006, 2007, 2008, 2009a) applied to the lateral head of the gastrocnemius of the right leg. Biopsies were taken 3 cm apart in a proximal to distal fashion, along an anatomically located muscle mid belly plane under local anesthetic (2% lidocaine hydrochloride). The fascia of the muscle was specifically avoided (Trappe et al., 2013). Disposable manually primed biopsy needle guns were utilized (12 × 16, Disposable Monopty Core Biopsy Instrument, Bard Biopsy Systems, USA). Samples collected (20–30 mg) were immediately frozen in liquid nitrogen (−196°C) and stored at −80°C for later analysis. Serial biopsies separated by 3 cm have been previously demonstrated not to provoke stress proteins in residual tissue (Khassaf et al., 2001). Muscle was obtained at PrT, 15PoT, and 120PoT (Figures 1, **3**).

Muscle samples were later ground under liquid nitrogen to remove non-muscle (i.e., adipose, connective) tissue prior to homogenization with a sonicator (T10 Basic, IKA, ThermoFisher Scientific, Loughborough, UK) on ice in 1 mL TRIzol reagent, followed by a 10 min incubation period on ice, in preparation for RNA extraction.

#### RNA extraction blood and muscle samples

RNA was extracted utilizing a previously validated (Chomczynski and Sacchi, 1987) technique that has been utilized specifically for HSP assessment *in vivo* (Tuttle et al., 2015; Gibson et al., 2015a,b; Mee et al., 2016). Briefly, chloroform (Sigma Aldrich, Dorset, UK) was added to (200 μL for muscle; 40 μL for leukocytes) samples suspended in TRIzol reagent, then vortexed and left to incubate on ice for 10 min prior to centrifugation at 17,000 × *g* for 15 min at 4°C. The aqueous phase was carefully aspirated and equal volume of ice-cold 2-propanol (Sigma Aldrich, Dorset, UK) was added before a 15 min incubation period on ice and subsequent centrifugation at 17,000 × g for 15 min at 4°C. The supernatant was removed and the sample was washed with (1 mL for muscle; 100 μL for blood) ice-cold 75% ethanol (Sigma Aldrich, Dorset, UK) ahead of centrifugation at 5,400 × g for 8 min at 4°C. Two additional ethanol washes were performed. Remaining ethanol was aspirated and the pellet was allowed to air dry for 15 min prior to the addition of 50 μL of RNA storage solution (Invitrogen, Paisley, UK). RNA quantity and quality were assessed optically at a density of 260 nm and ratios of 260/280 and 260/230, respectively, utilizing spectrophotometry (Nanodrop 2000c, ThermoFisher Scientific, Loughborough, UK). Only samples with a 260:280 ratio of between 1.9 and 2.15 were carried forward for reverse transcription and PCR amplification detailed below.

#### One-step reverse transcription quantitative polymerase chain reaction (RT-qPCR)

Primers (Table 2) were designed using primer design software (Primer Quest and Oligoanalyzer-Integrated DNA technologies) and have been recently utilized elsewhere (Tuttle et al., 2015; Gibson et al., 2015a,b; Mee et al., 2016). During primer design, in line with (Tuttle et al., 2015), sequence homology searches were performed against the GenBank database to ensure the primers matched the gene of interest. Primers were designed to span exon-intron boundaries and avoided three or more guanine-cytosine bases within the last five bases at the 3' end of primer to avoid nonspecific binding. Further searches were performed to ensure primers did not contain secondary structures and intermolecular or intramolecular interactions (hairpins, self-dimer, and cross dimers), which can inhibit product amplification.

**Table 2:** Primer sequences used in One-step reverse transcription quantitative polymerase chain reaction.

**Target gene**	**Primer sequence (5′-3′)**	**Reference sequence no**.	**Amplicon length (bp)**	**GC% content**
B_2_ microglobulin	Forward: CCGTGTGAACCATGTGACT	NM_004048	19	52.63
	Reverse: TGCGGCATCTTCAAACCT		18	50.00
HSP 72	Forward: CGCAACGTGCTCATCTTTGA	NM_005345	20	50.00
	Reverse: TCGCTTGTTCTGGCTGATGT		20	50.00
HSP32	Forward: CAGCAACAAAGTGCAAGAT	NM_002133	19	42.11
	Reverse: CTGAGTGTAAGGACCCATC		19	52.63

Relative Hsp mRNA expression was then quantified using RT-qPCR adhering to the method adopted by Tuttle et al., (2015). Reactions (20 μL) containing 10 μL of SYBR Green RT-PCR Mastermix (Quantifast SYBR Green kit; Qiagen, Manchester, UK), 0.15 μL of forward primer, 0.15 μL of reverse primer, 0.2 μL of reverse transcription mix (Quantifast RT Mix, Qiagen), and 9.5 μL sample (70 ng RNA/μL) were prepared using the Qiagility automated pipetting system (Qiagen). Each reaction was amplified in a thermal cycler (Rotorgene Q, Qiagen) and involved reverse transcription lasting 10 min at 50°C and a transcriptase inactivation and initial denaturation phase lasting 5 min at 95°C. The PCR reaction then followed with a denaturation step lasting 10 s at 95°C and a primer annealing and extension stage lasting 30 s at 60°C repeated for 40 cycles. Fluorescence was measured following each cycle as a result of the incorporation of SYBR Green dye into the amplified PCR product. Melt curves (50–95°C; Ramp protocol, 5-s stages) were analyzed for each reaction to ensure only the single gene of interest was amplified.

The relative quantification of mRNA expression for each sample was assessed by determining the ratio between the cycle threshold (CT)-value of the target mRNA and the CT-values for β2-microglobulin. Fold change in relative mRNA expression was calculated using the 2-ΔΔCT method (Schmittgen and Livak, 2008).

### Statistical analyses

All data was analyzed using the statistical software package IBM SPSS version 19.0 (SPSS Inc., Chicago IL, USA). Prior to any performance of inferential statistics, descriptive tables and graphical methods (Q–Q plots and scatter plots) were utilized to check for statistical assumptions with all data presented deemed to be normally distributed. A one-way ANOVA was used to assess for statistical differences between participants' anthropometric data. One-way repeated measures ANOVA was utilized to establish significant differences between hemoglobin saturation and HR during the hypoxic intervention period. Sphericity was assumed for all repeated measures analysis. Linear mixed models were used to identify significant group × time interactions in the remaining dependent variables across all groups. In the event of a significant *F* ratio for both linear mixed models and one-way repeated measures ANOVA, the *post-hoc* test Sidak was used to locate significant pairs. The most suitable covariant model was decided using the difference in −2 restricted log likelihood figures and the number of parameters of the two models tested against the χ^2^ critical statistic (Field, 2013). Furthermore, residuals were checked for normality and homogeneity of variance using Q-Q plots and scatter plots, respectively, and were considered plausible for all dependant variables. Statistical significance was assumed at *p* < 0.05. Finally, Cohen's effect sizes for independent means were calculated utilizing the formula outlined by Cohen and were established as: small (*d* = 0.2), medium (*d* = 0.5), and large (*d* = 0.8) effects (Cohen, 1992).

## Results

A significant difference was observed in age [*F*(_2, 15)_ = 4.36, *p* = 0.032] between IPC_*I*_ and CON (*p* = 0.032). No other significant differences (*p* ≥ 0.34) were noted in participant demographics (Table 1).

**Figure 2:**
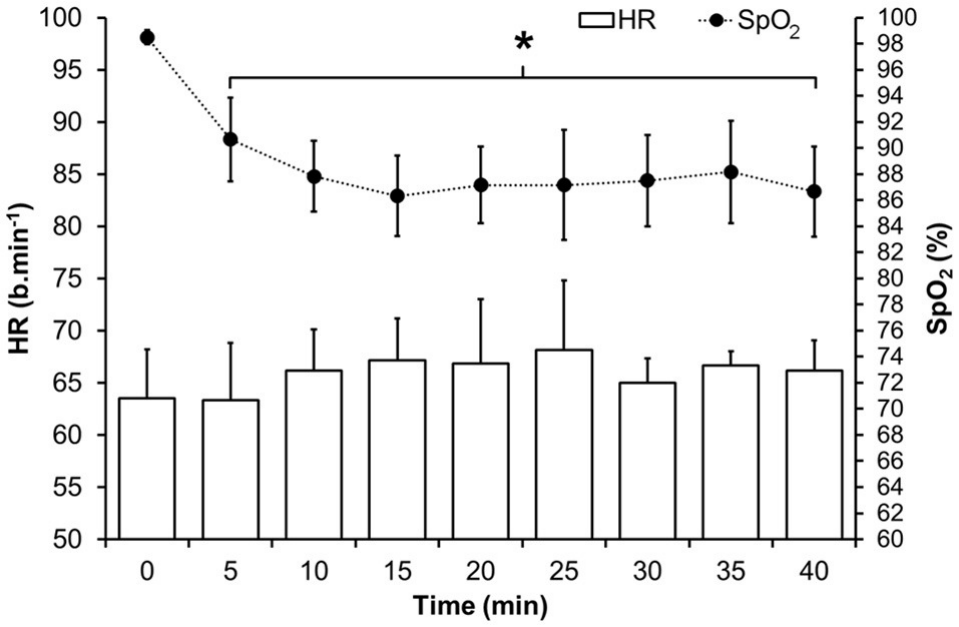
Mean (*SD*) HR and oxyhaemoglobin saturation during the hypoxic intervention in HPC_*I*_. ^*^Indicates significant difference (*p* < 0.05) vs. baseline values.

A significant main effect displayed a decrease in hemoglobin saturation [*F*_(8, 40)_ = 17.331, *p* < 0.001] between baseline and all subsequent time points (*p* < 0.05) in the HPC_I_ intervention. However, there was no significant main effect [*F*_(8, 40)_ = 1.130, *p* = 0.365] in HR noted by the same exposure (Figure 2).

**Figure 3:**
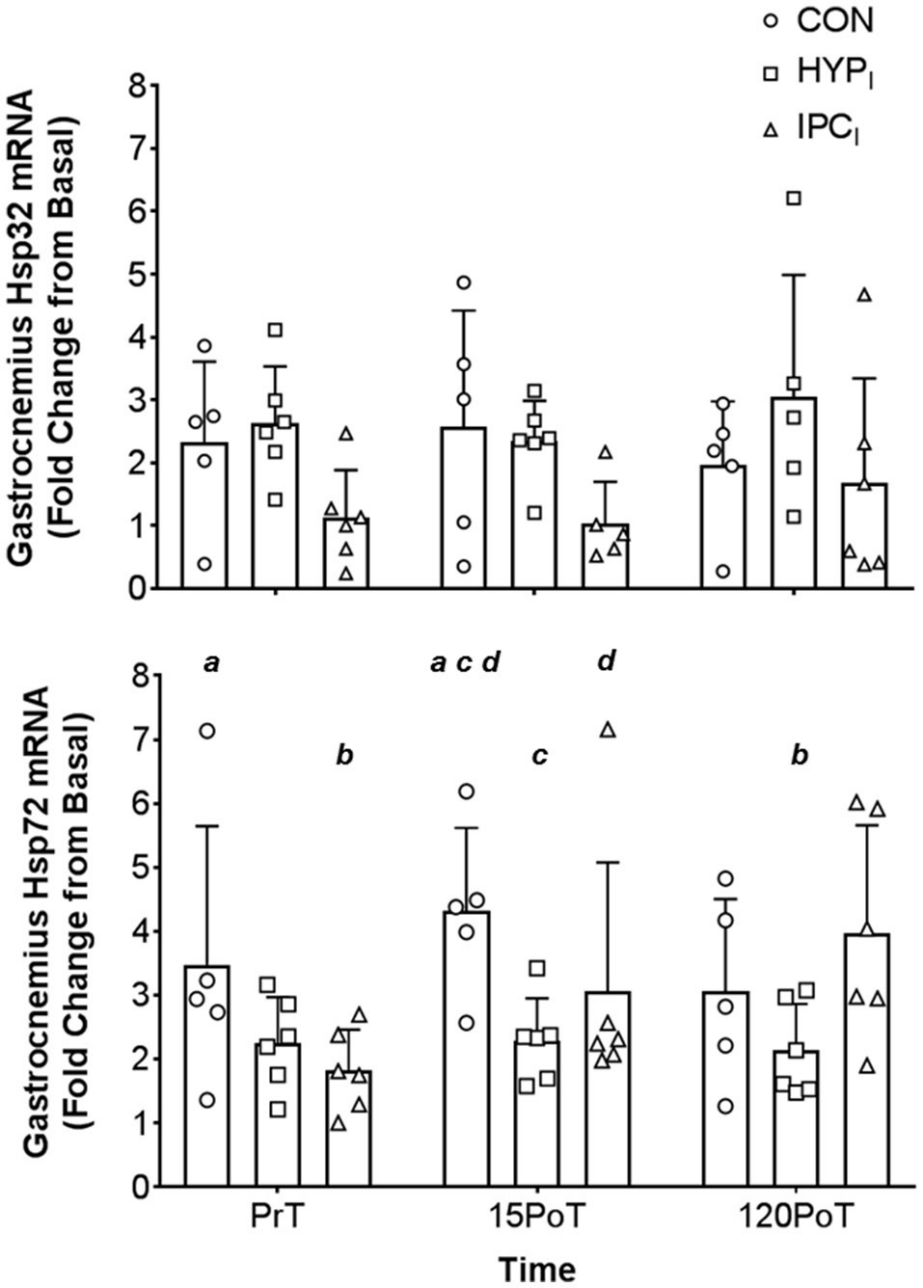
Mean (*SD*) gastrocnemius Hsp32 mRNA **(top)** and Hsp72 mRNA **(bottom)** expression. Gastrocnemius samples were obtained from CON (*n* = 5), HPC_*I*_ (*n* = 6), and IPC_*I*_ (*n* = 6) immediately pre-tourniquet application (PrT), 15 min post-tourniquet removal (15PoT), and 120 min post-tourniquet removal (120PoT). All experimental samples were expressed relative to a laboratory control gastrocnemius sample (from Basal). Letters (a, b, c, d) denote significant differences between corresponding letter (*p* < 0.05).

Significant group × time interaction effects (*F* = 3.058, *p* = 0.048) were observed in muscle Hsp72 relative gene expression. There was an increase between time-points PrT and 15PoT (95% CI −3.771, −0.124; *p* = 0.035) in CON displaying a large effect size (1.44). Also, between PrT and 120PoT, a pronounced 116% increase (95% CI −3.779, −0.400; *p* = 0.014) was noted in IPC_*I*_ producing a large ES (1.59). Furthermore, when compared to CON at 15PoT muscle Hsp72 was lower in HPC_*I*_ (95% CI 0.634, 3.934; *p* = 0.007) and IPC_*I*_ (95% CI 0.675, 4.114; *p* = 0.006), both demonstrating large effect sizes (1.90 and 2.19, respectively) (Figure 3).

There were no significant (*p* > 0.05) group × time interaction effects for gastrocnemius Hsp32 (*F* = 0.147, *p* = 0.961) (Figure 3) leukocyte Hsp72 (*F* = 1.195, *p* = 0.347), leukocyte Hsp32 (*F* = 1.406, *p* = 0.244), PC (*F* = 0.681, *p* = 0.707), or GSH/GSSG (*F* = 1.959, *p* = 0.105) (see Table 3).

**Table 3:** Mean (*SD*) circulatory markers of redox disturbance and stress protein expression.

**Measure**	**Basal**			**PoI**			**PrT**			**15PoT**			**120PoT**		
	**CON**	**HPC_I_**	**IPC_I_**	**CON**	**HPC_I_**	**IPC_I_**	**CON**	**HPC_I_**	**IPC_I_**	**CON**	**HPC_I_**	**IPC_I_**	**CON**	**HPC_I_**	**IPC_I_**
Leukocyte Hsp72 (relative fold change from basal)	1.46 (0.42)	1.29 (0.39)	1.72 (0.54)	1.46 (0.55)	1.23 (0.44)	1.31 (0.17)	1.43 (0.41)	1.45 (0.36)	1.31 (0.35)	1.44 (0.43)	1.57 (0.31)	1.33 (0.14)	1.23 (0.44)	1.35 (0.27)	1.35 (0.21)
Leukocyte Hsp32 (relative fold change from basal)	1.34 (0.43)	1.08 (0.24)	1.47 (0.55)	1.09 (0.33)	1.14 (0.24)	1.23 (0.40)	0.86 (0.14)	1.10 (0.28)	1.29 (0.29)	0.86 (0.28)	1.24 (0.27)	1.17 (0.33)	1.04 (0.45)	1.02 (0.41)	1.08 (0.38)
Protein Carbonyl (nmol·mL^−1^)	0.56 (0.14)	0.56 (0.19)	0.69 (0.24)	0.54 (0.16)	0.42 (0.13)	0.61 (0.13)	0.64 (0.13)	0.58 (0.08)	0.69 (0.21)	0.65 (0.18)	0.60 (0.24)	0.56 (0.15)	0.65 (0.32)	0.69 (0.15)	0.63 (0.21)
Reduced/oxidized glutathione ratio	23.0 (9.1)	31.4 (12.2)	22.7 (8.6)	24.6 (9.6)	29.3 (5.4)	28.9 (7.4)	27.1 (11.6)	23.9 (7.3)	18.6 (7.6)	27.6 (10.7)	20.5 (3.6)	23.1 (8.7)	23.0 (7.7)	22.4 (2.2)	20.5 (9.6)

*Measurements were evaluated at basal, immediately post-intervention (PoI), immediately pre-tourniquet application (PrT), 15 min post-tourniquet removal (15PoT), and 120 min post-tourniquet removal (120PoT)*.

## Discussion

In agreement with the presented hypothesis it is a novel experimental finding that both IPC_I_ and HPC_I_ primed the localized (gastrocnemius) HSP system producing a blunted response in Hsp72 mRNA post (15PoT) 30 min TKR like femoral blood flow occlusion (termed simply occlusion from this point forward) compared to CON (Figure 3). However, changes in local Hsp32 were not seen (Figure 3), contrary to the stated hypothesis, this lack of response may be indicative of minimal intramuscular OS within the gastrocnemius following 30 min of occlusion. This amelioration in Hsp72 response (15PoT) was seen without systemic changes in OS (PC and GSH:GSSG; Table 3) and leukocyte Hsp32, in agreement with the stated hypothesis.

HSPs are up-regulated in response to a variety of stressors relevant to IR and HReox mediated *in vivo* stress, including OS, hypoxia, and ischemia (Kalmar and Greensmith, 2009; Morton et al., 2009b; Gibson et al., 2017). Elevated localized Hsp72 mRNA in CON at 15PoT (+84 ± 78%) indicates that the occlusion stressor was sufficient to potentiate initiation of the heat shock response (Theodorakis et al., 1999; Noble et al., 2008), whilst amelioration of this response (indicative of early phase IPC tissue protection; Bushell et al., 2002a,b; Loukogeorgakis et al., 2005) was seen when occlusion was preceded by IPC_*I*_ (+19 ± 35%) or (HPC_*I*_ +7 ± 33%; Figure 3). This change in Hsp72 mRNA within CON was not confounded by the larger basal gene expression of one participant in this group at this time point. Amelioration of Hsp72 mRNA is not novel regarding efficacy of IPC to blunt the respective gene response post-reperfusion of occluded distal lower limb skeletal muscle (rat tibialis anterior) (Bushell et al., 2002a,b) however, blunting of the Hsp72 mRNA stress response *in vivo* via HPC_*I*_ within the presented paradigm is. It is ecologically relevant that the blunted response in HPC_*I*_is contained within a time frame whereby surgery may be performed i.e., up to 120PoTwith the time course of responses comparable to those observed in similar Hsp72 mRNA experiments (Febbraio et al., 2002; Tuttle et al., 2017). Mechanistically, this intervention mediated reduction in Hsp72 mRNA cannot be attributed to systemic reduction in OS (PC or GSH:GSSG; see Table 3) nor increased degradation of ROS-producing heme molecules by Hsp32 mRNA, as these outcome variables were unchanged within and between all conditions and time points (Table 3, Figure 3). However, the absence of localized measures of OS precludes inferences relative to a local to systemic difference in OS (although others have shown such a difference post knee surgery which utilized a tourniquet; Karg et al., 1997) mediating the local to systemic differential Hsp72 mRNA response at 15PoT. Given the high affinity for changes in OS to induce a HSP32 and Hsp32 mRNA heat shock response (Gozzelino et al., 2010; Taylor et al., 2012), and the absence of changes in Hsp32 mRNA locally (and systemically), it is likely local Hsp72 mRNA amelioration via the interventions at 15PoT is mechanistically distinct from OS—at least within the occlusion paradigm utilized within the present design.

IPC has been cited to diminish circulatory redox disturbances associated with tourniquet-induced IR stress following TKR surgery (Koca et al., 2011). This is in disagreement with the current study, whereby it was noted that systemic OS markers remained unchanged within and between conditions at all times points when assessing lipid peroxidation (malondialdehyde concentrations) via measurement of thiobarbituric acid-reactive substances (Koca et al., 2011). This particular method of assessing malondialdehyde lacks specificity (Powers et al., 2010b), as such, the changes noted may be due to methodical inaccuracies rather than experimental effect. Additionally, systemic OS may not be a reliable surrogate marker for an intracellular response as aforementioned. It is however plausible that the 30 min occlusion utilized by the present study did not induce sufficient stress to observe changes in systemic OS markers and leukocyte Hsp mRNA (Table 3). This occlusion duration is lower than that commonly used during TKR surgery (mean ± *SD*; 79.9 ± 12.7 min; Cheng et al., 2003). Therefore, it is expected that the OS would be greater during surgery thus the occlusion stressor in the present study is not completely externally valid. However, our pilot testing revealed a longer occlusion period was not tolerable *in vivo* by non-anesthetized humans thus precluding its implementation. In an exercise setting, variants of our IPC (de Groot et al., 2010; Bailey et al., 2012; Cruz et al., 2015; Kido et al., 2015; James et al., 2016; Sabino-Carvalho et al., 2016), and HPC (Lee et al., 2014; Turner et al., 2016; Chacaroun et al., 2017), have been implemented to induce positive physiological responses. These data reflecting positive responses in clinical paradigms (Landry et al., 1982; Tomai et al., 1999; Otani, 2008; Mateika et al., 2014; Verges et al., 2015; Baillieul et al., 2017). Our data present a novel contribution to the area demonstrating the potential facilitative role HSPs have in response to OS.

Early phase IPC protection is temporally aligned to a 1–2 h window (occlusion occurred within this window) post IPC, whilst late phase IPC protection commences ~24 h post IPC and has a window of effect between ~24 and ~72 h post IPC. This late phase protection is dependent on the induction of protective proteins (Loukogeorgakis et al., 2005) including HSP72 (Bushell et al., 2002a,b; Marber et al., 1993, 1995). HSP72 is thought to refold sub-lethally damaged proteins and diminish their interactions with viable proteins during repeated IR bouts (Marber et al., 1993), consequently conveying cellular protection. The cumulative IR signal in IPC_*I*_ [i.e., five distinct IR stressor bouts; intervention (four IR cycles) and occlusion (one 30 min IR cycle)] compared to HPC_I_ [occlusion (one 30 min IR cycle) preceded by interventional HReox] is greater. Therefore, intervention activated IR preconditioning specific biological processes may have been exacerbated (particularly IR mediated cyclical increases in bradykinin and adenosine; Loukogeorgakis et al., 2005) by the subsequent occlusion IR stress and may underpin the enhanced gastrocnemius Hsp72 mRNA transcription seen at 120PoT in IPC_I_, a response absent in HPC_I_. This could be an early transcriptional level initiation of late phase IPC, within HPC_I_, to which HSP72 protein translation is central (Loukogeorgakis et al., 2005). Therefore, this enhanced Hsp72 mRNA transcription at 120PoT could be interpreted as a priming mechanism for late phase IPC HSP72 translation within IPC_I_, a response absent within HPC due to a lack of sufficient IR signal. In support of this postulation it has been eloquently shown elsewhere that remote limb IPC within humans results in cumulative up-regulation of peptides, with the magnitude of increase dependent on the number of discrete IR bouts (Hepponstall et al., 2012). Future research should examine the accumulative and temporally ordered effects (in line with recent attempts within a rat model; Kocman et al., 2015) of IPC (IR) and HPC (HReox) conveyed preconditioning *in vivo* within humans. A specific emphasis should be placed on adenosine, bradykinin, extracellular signaling proteins (ERK1/2, AKT; Winter et al., 2016) and relevant gene transcription and translation relative to the temporally ordered protective effects (i.e., early and late phase IPC tissue protection; Loukogeorgakis et al., 2005). The lack of such an approach is a limitation of the present study.

Prior research has shown that both acute hypoxic exposure (Taylor et al., 2010a) and IPC (Konstantinov et al., 2004) can stimulate leukocyte HSP72, which is in contrary to the data presented here (Table 3). Indeed in support of an association between OS and HSP responses antioxidant elevations are associated with leukocyte Hsp72 reductions (i.e., a lesser necessity for HSP72 transcription), thus OS necessitating HSP transcription could increase Hsp72 in leukocytes (Simar et al., 2012). Likewise antioxidant supplementation (500 IU.day^−1^ RRR-a-tocopherol for 8 days) attenuates the change in leukocyte Hsp72 mRNA after exhaustive exercise (Niess et al., 2002). The extended 75 min hypoxic exposure (approximately the same 14.3% O_2_ as used within the present study) utilized previously (Taylor et al., 2010a), would have induced a more substantial OS stimulus for the induction of Hsp72, compared to the 40 min exposure utilized within HPC_I_ in the present study. Given the very nature of IPC, and the lack of a change in circulating markers of OS, it is possible that the leukocytes were unable to experience the necessary fluctuations in OS that occurred locally within the skeletal muscle undergoing biopsy. As such it may be an experimental artifact associated with IPC alone that is responsible for the lack of Hsp72 mRNA responses in leukocytes rather than a mechanistic one. Utilizing a multi-gender sample and gene array analysis to assess Hsp72 (Konstantinov et al., 2004), with the former known to influence stress mediated changes in HSP (Paroo et al., 2002; Morton et al., 2009a) and the latter known to both over and underestimate gene transcript response (Feldman et al., 2002), may also explain some of the disparity in Hsp72 mRNA results.

Given that systemic OS was unchanged within and between all conditions (in line with previous data; Karg et al., 1997), it is likely that this absence of the potent OS stimuli (particularly ROS producing free-heme molecules) underpinned the lack of leukocyte Hsp32 mRNA response (Fehrenbach et al., 2003a) (Table 3). Although local OS markers were not obtained, absence of muscle Hsp32 mRNA change suggests only minimal (if any) disturbances in OS locally. To determine whether the 30 min occlusion was sufficiently stressful to induce intramuscular OS, future work should measure markers of this within the target tissue. To the authors' knowledge measurement of leukocyte and skeletal muscle Hsp32 mRNA within the presented *in vivo* occlusion paradigm is novel, with previous rodent model data lacking external validity to the presented TKR specific rationale (particularly time course). Rodent model data indicates that with five cycles of IPC (compared to the present study), muscle Hsp32 protein displays a 2-fold increase with glutathione remaining unchanged, albeit only assessed 48 h post IPC (Badhwar et al., 2004). The stable Hsp32 mRNA-values could be a fiber type specific response, with type I muscle fibers shown to readily express HSP32 in comparison to a blunted response in type II fibers (Vesely et al., 1999). The lateral head of the gastrocnemius consists of equal proportions of both fiber types (Edgerton et al., 1975). Therefore, the response observed in the present study may only be proportional to the percentage of type II fibers in the biopsied muscle. A lack of fiber type characterization within the gastrocnemius samples within the present study is an experimental limitation that should be addressed by future research designs.

HPC has been shown to blunt IR mediated tissue damage in animal models (Beguin et al., 2005; Berger et al., 2010), however, to the author's knowledge, no previous studies have performed HPC in humans prior to limb-tourniquet application and subsequent IR stress. HPC is thought to confer cellular protection through similar mechanisms to IPC, essentially hormesis from appropriate protein accumulation (the precise mechanism/stimuli for such accumulation are not robustly described *in vivo*). The present data provides provisional evidence that *in vivo* HPC prior to IR stress provides similar reductions in localized cellular stress as an established IPC model. Interestingly, IPC has preliminary *in vivo* evidence supporting its use to reduce post-operative pain following TKR surgery (Memtsoudis et al., 2010), although some equivocal evidence is also present (Memtsoudis et al., 2014). Practically, IPC involves close monitoring to ensure correct ischemic periods (typically within theater), yet the HPC_*I*_ protocol used here would not require this human resource as the inhalation of hypoxia is continuous, thus potentially allowing HPC to take place during surgical preparation (perhaps on the ward utilizing methods described earlier). It should be noted that previous work has utilized intermittent hypoxia as a preconditioning strategy, future experimental designs should make comparisons between continuous and hypoxic interventions as previously utilized (albeit with differing clinical applications) in animals (Beguin et al., 2005) and humans (Lyamina et al., 2011; Chacaroun et al., 2017), to determine which elicits the most desirable preconditioning responses. From a practical perspective an intermittent approach may be less practical to administer in clinical setting if using the current equipment i.e., it would require manually switching between hypoxic and normoxic air. However, further experimentation is required to demonstrate whether localized reductions in cellular stress (Hsp72 mRNA) from HPC_I_ (as also seen in IPC_I_) can convey the same positive effects (reduced pain, accelerated wound healing, reduced length of hospital stay, as raised within the Introduction and Discussion sections) attributed to IPC, within externally valid models related to TKR like knee surgery which utilizes a tourniquet. Indeed, concerns could be raised to the safety of exposing elderly patients (population most likely to receive TKR surgery for example) to acute hypoxia. However, research has demonstrated that an elderly population with a high prevalence of cardiovascular disease tolerated hypobaric hypoxic remarkably well (Levine et al., 1997). Given that exercise and heating initiates increases in Hsp72 mRNA (Fehrenbach et al., 2001; Gibson et al., 2015a,b), in a temperature dependent manner (Gibson et al., 2016), passive heating [which has also been shown to increase Hsp72 mRNA; Horowitz et al., 1997; Maloyan et al., 1999] may also provide a viable preconditioning strategy that may be applied on the ward prior to surgery. In addition to the application presented (i.e., reperfusion) both HPC and IPC appear to initiate cellular responses (HSP increases) which are likely beneficial in across stimuli e.g., exercise, hypoxia, heat, though this is yet to be experimentally elucidated.

Although adequately powered it is important that the present data and postulations relative to the use of HPC are viewed relative to this sample size and preliminary nature of the experimental findings/design. It has been previously observed there can be high variability between participants with regards to basal and stress-mediated values of these measures, this variation based upon individual differences in physiological profile (Fehrenbach et al., 2000a,b, 2003a; Bruce et al., 2003) which require consideration relative to the presented data and discussion of the application of HPC vs. IPC prior to surgery. These reasons may explain the high basal Hsp72 mRNA content in one CON group participant in the present study. As such further work with populations displaying more diverse phenotypes are warranted. In additions to experimental limitations acknowledged throughout the discussion, given many antioxidants are procured naturally from the diet, all with varying half-lives, this could have influenced OS related outcome variables within the present study (Powers et al., 2010a). Although every effort was made to control this (standardized morning and afternoon meal), it is extremely challenging to control participants' diet over a long period of time, while ensuring continued participation. Therefore, the lack of change in OS markers could have partly been due to dietary variation.

In summary, it can be seen that a bout of HPC_I_ primed the HSP system thus bestowing localized cellular protection to tourniquet IR mediated stress induced via an ecologically valid TKR model. Furthermore, HPC_I_ provided similar levels of cellular protection to IPC_I_, thus providing a novel framework for the use of HPC_I_ to convey cellular protection in light of a subsequent IR related stressor.

## Author contributions

JB participated in the study conception, methodological optimization, data collection, sample and statistical analysis and manuscript drafting and revisions. OG participated in the statistical analysis and manuscript drafting and revisions. JT aided in data collection, sample and statistical analysis and manuscript revision. BC assisted with statistical analysis and manuscript revision. OP, JP, SG, CK, NG, FR, ZO, AS, and SN contributed to the study conception and optimization of muscle sample collection. LT participated in study conception, optimization of muscle sample collection, manuscript drafting and revision. All authors have read and approved the final manuscript.

## Availability of data and materials

The datasets during and/or analyzed during the current study available from the corresponding author on reasonable request.

### Conflict of interest statement

The authors declare that the research was conducted in the absence of any commercial or financial relationships that could be construed as a potential conflict of interest.

## Abbreviations

CON: control experimental condition
HSP: heat shock protein
Hsp: heat shock protein gene transcript
HPC: hypoxic preconditioning
HPC_I_: hypoxic preconditioning experimental condition
IPC: ischemic preconditioning
IPC_I_: ischemic preconditioning experimental condition
IR: ischemic reperfusion
HReox: hypoxia/reoxygenation
RT-qPCR: one-step reverse transcription quantitative polymerase chain reaction
OS: oxidative stress
OS: oxidized glutathione
PC: protein carbonyl
ROS: reactive oxygen species
GSH: reduced glutathione
TKR: total knee replacement surgery.
